# Transposed-word effects in speeded grammatical decisions to sequences of spoken words

**DOI:** 10.1038/s41598-022-26584-2

**Published:** 2022-12-21

**Authors:** Sophie Dufour, Jonathan Mirault, Jonathan Grainger

**Affiliations:** 1 Laboratoire Parole et Langage, Aix-Marseille Université, CNRS, LPL, UMR 7309, 5, avenue Pasteur, 13100 Aix-en-Provence, France; 2 Laboratoire de Psychologie Cognitive, Aix-Marseille Université & CNRS, Marseille, France; 3 Institute for Language, Communication, and the Brain, Aix-Marseille Université, Aix-en-Provence, France; 4 Pôle pilote AMPIRIC, Institut National Supérieur du Professorat et de l’Éducation (INSPÉ), Aix-Marseille Université, Aix-en-Provence, France

**Keywords:** Psychology, Human behaviour

## Abstract

We used the grammatical decision task (a speeded version of the grammaticality judgment task) with auditorily presented sequences of five words that could either form a grammatically correct sentence or an ungrammatical sequence. The critical ungrammatical sequences were either formed by transposing two adjacent words in a correct sentence (transposed-word sequences: e.g., “The black was dog big”) or were matched ungrammatical sequences that could not be resolved into a correct sentence by transposing any two words (control sequences: e.g., “The black was dog slowly”). These were intermixed with an equal number of correct sentences for the purpose of the grammatical decision task. Transposed-word sequences were harder to reject as being ungrammatical (longer response times and more errors) relative to the ungrammatical control sequences, hence attesting for the first time that transposed-word effects can be observed in the spoken language version of the grammatical decision task. Given the relatively unambiguous nature of the speech input in terms of word order, we interpret these transposed-word effects as reflecting the constraints imposed by syntax when processing a sequence of spoken words in order to make a speeded grammatical decision.

## Introduction

Recent research has shown that spoken word recognition is relatively robust to small changes in the order of phonemes in words. As a result, speech input like [kat] not only provides support for the corresponding lexical representation *cat* but also for the lexical representation that contains the same phonemes in a different order *tack*^1–3^. Furthermore, it has been shown that nonwords like /ba**ks**ɛt/ created by transposing two phonemes of the real word /*ba****sk***ɛ*t*/ are perceived as being more similar to the base word /*ba****sk***ɛ*t*/ than nonwords like /ba**pf**ɛt/ created by substituting two phonemes of the same words^4,5^. These observations clearly argue for some flexibility in phoneme-order encoding during spoken word recognition (see^6^, for a proposition of how this might be achieved).

Given that phonemes occur in rapid succession in spoken words, it could be argued that transposed-phoneme effects are principally the result of a noisy bottom-up assignment of phonemes to their positions in a word, much like the assignment of letters to letter-in-word position in the overlap model^7^. In the present work we move up to the scale of words and sentences (as opposed to phonemes and words), where it might be expected that ambiguity in word order would be greatly reduced compared with phoneme order. That is, given the rapidity with which individual words are processed, noisy auditory input might well affect the bottom-up association of phonemes to their positions in a string of phonemes. We suspect that this bottom-up noise will be greatly reduced when examining transposed-word effects as opposed to transposed-phoneme effects. In any case, the simple question to be answered by the present work is: can one observe transposed-word effects during spoken sentence processing in the same way as has been previously reported for reading, and in a manner analogous to the transposed-phoneme effects seen in spoken word recognition?

Transposed-word effects with written materials have been reported in the field of linguistics, but under testing conditions quite different from the conditions typically used to investigate transposition effects in experimental psychology (e.g., where response times (RTs) are typically the main dependent variable). This prior work does, however, provide an important theoretical background for understanding the role of word order information in sentence comprehension. At an empirical level, this research has demonstrated that, given the goal to understand / interpret linguistic input, adult humans are capable of recovering from various types of error including errors in word order (see^8^, for a review, and for a specific investigation of word order errors using fMRI). One influential account of how readers can recover from such errors is the noisy channel model proposed by Gibson, Levy, and colleagues (e.g.,^9–11^). The general idea is that noisy input to the syntactic parser provides support for what has been referred to as “good-enough” representations for language comprehension (e.g.,^12^; see also the “lossy-context surprisal” model of Futrell et al.^13^). Such good-enough or incomplete representations of the input would promote sentence interpretability in the face of ungrammaticality caused, for example, by changes in word order. Furthermore, as noted by Mollica et al.^8^, not all languages apply strict word-order rules, and given this, one other means to recover from incorrect word order might be via an autonomous semantic analysis of the sequence of words—an analysis that would be more tolerant to syntactically illegal word order (e.g.,^14^).

Returning to investigations within the field of experimental psychology, one study in particular forms the starting point of the present work. That is the study by Mirault et al.^15^ using sequences of written words presented simultaneously in a normal text format. That study introduced two novelties to reading research: (1) a speeded version of grammaticality (or well-formedness) judgments, referred to as the grammatical decision task in our later work (in analogy with the lexical decision task—e.g.,^16^); and (2) the introduction of a baseline control condition against which performance to transposed-word sequences could be compared. Concerning the first point, we readily acknowledge the vast area of research in linguistics where grammaticality or well-formedness judgments have been used to investigate sentence processing (e.g.^17^,), but we stress one key difference in our approach—that participants are instructed to respond as rapidly and accurately as possible. This is the sentence-level equivalent of the most widely used task to study single word recognition—the lexical decision task (a speeded binary decision task). Crucially, theoretical psychologists have gone to great extents to develop mathematical models of speeded decision making (e.g.,^18^). Research using the lexical decision task has benefitted from such theoretical support (e.g.,^19^;^20^), and we expect that the same will hold for research using the grammatical decision task (see^21^, for preliminary evidence).

Concerning the second point, our study goes beyond prior research using un-speeded grammaticality/well-formedness judgments not only by the speeded nature of the task, but also by enabling a comparison of performance across carefully matched ungrammatical sequences. The choice of control sequences was described in detail in our prior work^15^ and is re-described here for convenience. The critical stimuli in our experiment are the ungrammatical sequences. The grammatically correct sentences were added for the purpose of the grammatical decision task. We created two types of ungrammatical sequence: (1) sequences formed by transposing two adjacent words in a correct sentence (and neither of the two words could be at the first or last position in the sequence), and (2) matched ungrammatical sequences that could not be resolved into a correct sentence by transposing any two words. The matching was done using pairs of grammatically correct base sentences (e.g., The black dog was big/The black dog ran slowly), that were not tested in the experiment. These were used to generate the transposed-word ungrammatical sequences (e.g., The black was dog big / The black ran dog slowly) and the control ungrammatical sequences (e.g., The black was dog slowly / The black ran dog big), such that the same words in the same position appeared in both types of ungrammatical sequence (see Table 1 for a summary of the conditions).

**Table 1:** English examples illustrating how the critical ungrammatical test sequences used in the Experiment were constructed from quadruplets of base sequences of five words that could either form a correct sentence (grammatical) or not (ungrammatical). Examples are in English for convenience, whereas the experiment was in French.

**Base sequences**
Grammatical: The black dog was big/The black dog ran slowly
Ungrammatical: The black dog ran big/The black dog was slowly
**Test sequences**
Transposed-word: The black was dog big/The black ran dog slowly
Control: The black ran dog big / The black was dog slowly

The key finding from our prior research using this specific procedure and methodology^15^ was that participants found it harder (longer RTs and more errors) to reject the transposed-word sequences as being ungrammatical compared with the control sequences. Mirault et al. interpreted this finding as reflecting partially parallel processing of words during written sentence comprehension (the evidence at present suggests that information concerning the syntactic category^22^ and the semantic content^23^ of words can be processed in parallel) and the noisy bottom-up assignment of word identities (i.e., orthographic word forms) to positions in the sequence, combined with top-down constraints from sentence-level syntactic representations (see^24^;^25^ for replications and extensions of this work).

Contrary to the parallel processing account of transposed-word effects (see^26^ for a summary), recent work from our group^27^ and work in Chinese^28^ has shown that transposed-word effects can be observed with written materials even when the words are presented sequentially (one after the other at the same central location) using rapid serial visual presentation (RSVP: 250 ms per word in the Liu et al.^28^ study, and 300 ms per word in the Mirault et al.^27^ study). However, the pattern that has systematically emerged from this research is that contrary to effects obtained with parallel word presentation, transposed-word effects obtained under serial presentation only emerge in error rates and not in RTs. Given that serial word presentation greatly reduces word position uncertainty in bottom-up processing, Mirault et al.^27^ interpreted this pattern as reflecting the impact of top-down syntactic constraints on word-order encoding in the absence (or reduction) of bottom-up positional noise.

In the present study we examined transposed-word effects in spoken language comprehension in order to provide a test of top-down (interactive) influences on spoken sentence processing. Given the strictly sequential nature of word recognition during spoken language comprehension, plus the rather long durations of each word in typical speech it would appear unlikely that bottom-up processing could be noisy enough to create confusion about word order. Therefore, we reasoned that observing a transposed-word effect with sequences of spoken words would provide evidence in favor of syntactic constraints affecting the order in which the words are perceived. Note that this is the first study to examine transposed-word effects using the grammatical decision task (i.e., the speeded version of grammaticality judgments) with spoken word sequences, hence providing a crucial test of the viability of this task for future research on spoken language comprehension.

## Methods

### Participants

100 participants were recruited via the Prolific platform for on-line experiments. The number of participants was chosen to respect the number tested in the on-line study of Mirault et al.^15^, which was 94 (thus allowing for the possibility of a small number of exclusions). They reported to be native speakers of French and their reported age was between 18 and 62 years. Prior to the beginning of the experiment, participants provided informed consent and they were informed that the data would be collected anonymously. Participants received 8£ per hour in compensation.

### Materials

The stimuli and design were the same as in the Mirault et al.^15^ study and are summarized here for convenience. 40 pairs of grammatically correct sentences composed of five words (e.g., ton petit chat avait faim (*your little cat was hungry*); cette grande tasse est belle (*this big cup is beautiful*)) were used to generate the critical ungrammatical transposed-word and control sequences. The transposed-words sequences were created by simply transposing the third and fourth words in the correct sentences (e.g., ton petit avait chat faim); cette grande est tasse belle). Matched ungrammatical control sequences were created from the same set of pairs of correct sentences by first making them ungrammatical by switching the final words in each sentence (e.g., ton petit chat avait belle; cette grande tasse est faim. Then, the same words as in the transposed-word sequences were transposed (e.g., ton petit avait chat belle; cette grande est tasse faim) but contrary to the transposed-word sequences, transposing any two words in the control sequences could not generate a correct sentence. This further meant that across the whole set of critical stimuli the transposed-word sentences and control sentences contained the same words in the same position and that both types of sequence became ungrammatical at exactly the same position (see Table 1, for examples of the quadruplets that enabled this matching process). For the purpose of the grammatical decision task, 80 different grammatically correct sentences were constructed. The sequences were recorded using “text-to-speech” for French and with the female voice called “Denise” and digitized at a sampling rate of 44,100 Hz. The mean duration of the critical ungrammatical sequences was 1372 ms for both the transposed-word and the control sequences. Because the 80 ungrammatical transposed-word sequences and the 80 ungrammatical control sequences were created from the same 80 base sentences, two counterbalanced lists of stimuli were constructed to avoid repetition of the same words within a list and across the two types of ungrammatical sequence (transposed-word, control). Each list thus contained 40 transposed-word sequences and 40 control sequences, and 80 grammatically correct sentences.

### Procedure

The experiment was programmed using Labvanced software^29^. Participants were instructed to put on their headphones and adjust the volume to a comfortable sound level. A trial began with a centrally aligned fixation cross for a duration of 500 ms, followed by the auditory sentence at an average rate of 274 ms per word. For each word sequence, participants were asked to make a grammatical decision as quickly and accurately as possible by pressing the “right arrow” for grammatically correct sentences and the “left arrow” for ungrammatical sequences. After each response, a feed-back consisting in a green circle in case of a correct response and in a red circle in case of an incorrect response was presented for a duration of 500 ms. No mention was made of the word order manipulation, but the feedback provided after each trial clearly indicated that an “ungrammatical” response was expected on trials with a transposition. The order of presentation of word sequences was randomized for each participant. Participants were tested on only one experimental list and began the experiment with 8 practice trials.

### Ethical approval

The experiment was carried out in accordance with relevant guidelines and regulations and the experimental protocol was approved by the Comité de Protection des Personnes SUD-EST IV (No. 17/051).

## Results

**Figure 1:**
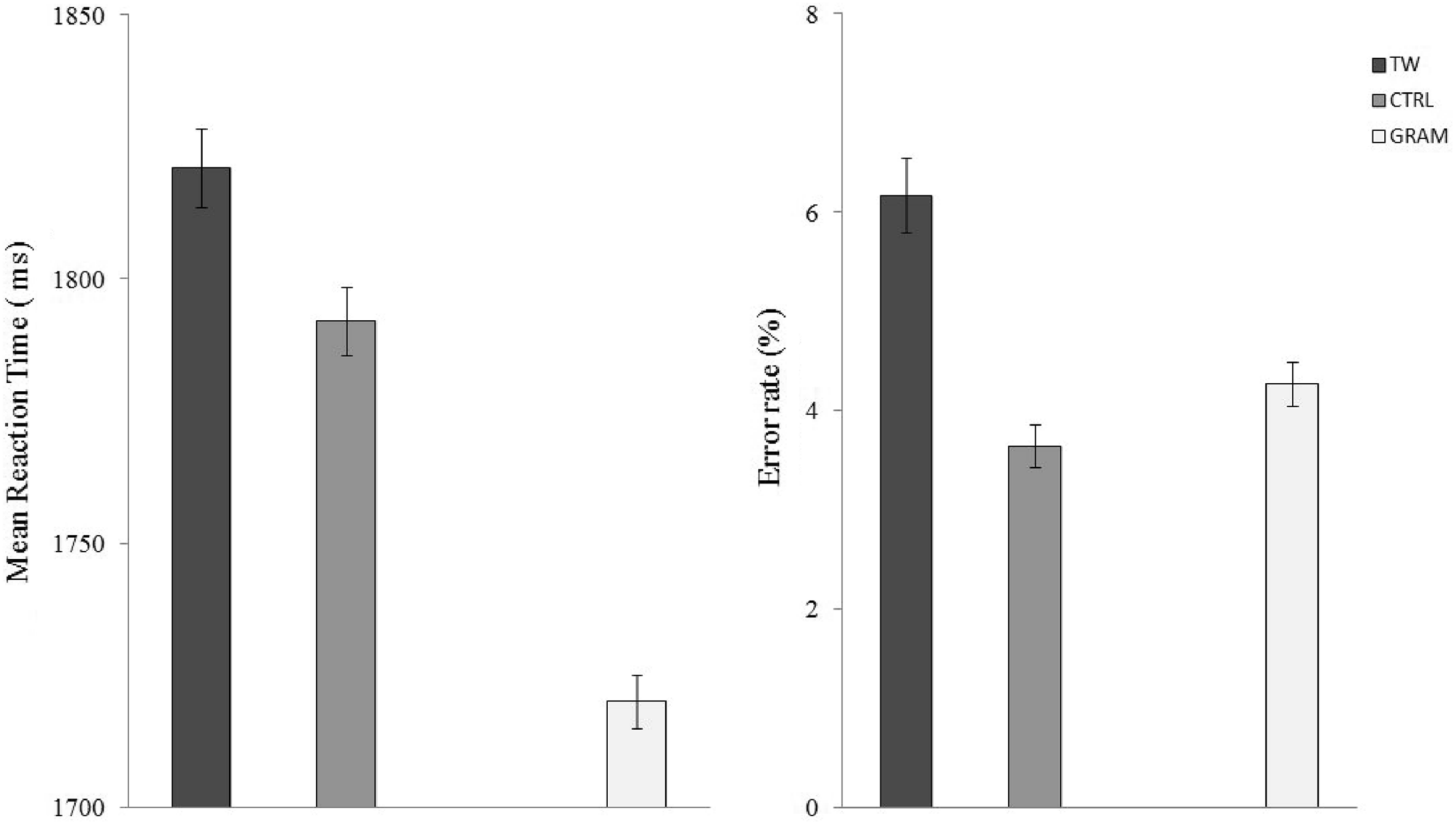
Mean Reaction Times (left) and error rates (right) for the transposed-word (TW) and control (CTRL) ungrammatical sequences. Error bars represent 95% confidence intervals. Means for the grammatically correct sentences (GRAM) are given for comparison.

Six participants were removed from the analyses. Three participants had an error rate greater than 30%, one had excessively long RTs (greater than 3000 ms), and for the two others, problems were encountered in the recording of RTs. The mean RT and percentage of correct responses in each condition are shown in Fig. 1.

RTs to the critical ungrammatical sequences were analyzed using linear mixed effects models with participants and items as crossed random factors, using R software and the lme4 package^30^. The RT analysis was performed on correct responses, thus removing 369 (4.91%) data points out of 7520. RTs greater than 5000 ms (0.25%) were considered as outliers and were also excluded from the analysis. For the model to meet the assumptions of normally-distributed residuals and homogeneity of variance, a log transformation was applied to the RTs^31^ prior to running the model. The model was run on 7133 data points. We tested a model with the variable Transposition (transposed-word, control) entered as fixed effect. The reference was the control condition, and we used the default (0, 1) coding. Note that the model failed to converge when random participant and item slopes were included^32^. Therefore, the final model only included random intercepts for participants and items.

The effect of Transposition was significant (*b* = 0.0138, *SE* = 0.0037, *t* = 3.76, *p* < 0.001). Participants were 28 ms slower at classifying transposed-word sequences as being ungrammatical in comparison to control sequences.

Response accuracy was analyzed using a mixed-effects logit model^33^ following the same procedure as for RTs. The effect of Transposition was again significant (*b* = − 0.6212, *SE* = 0.1167, *z* = − 5.32, *p* < 0.001). Participants made more errors in the transposed-word condition than in the control condition.

## Discussion

Prior research suggests that, given the goal to understand linguistic input, human adults are capable of recovering from various types of error, including errors in word order (see Mollica et al.^8^, for a review). The present study built on this prior work by examining whether one can observe a transposed-word effect in speeded grammatical decisions to sequences of spoken words in that same way that the effect has been observed with sequences of written words^15^, and similarly to the transposed-phoneme effects found in auditory lexical decision^4,5^. We observed that participants found it harder to classify a transposed-word sequence (e.g., “The white was cat big”) as being ungrammatical compared with the matched ungrammatical control sequences (e.g., “The white was cat slowly”). Although the transposed-word effect was numerically smaller than that seen with written materials, it was robust in both RTs and error rates.

In the Introduction, we summarized work with written words using the RSVP paradigm (i.e., sequential presentation of words) as opposed to the simultaneous presentation of words in the Mirault et al.^15^ study. RSVP is arguably closer to spoken language presentation, and one therefore might expect to see the same pattern of results in written RSVP and spoken word sequences. However, this is apparently not the case. Two written language studies have reported that under serial presentation conditions transposed-word effects are observed in error rates but not in RTs^27,28^. So, it remains to be explained why the full pattern of transposed-word effects in both RTs and accuracy is seen with spoken words (the present study), but not with sequentially presented written words.

One obvious difference between the RSVP paradigm and the present work concerns how familiar participants are with the presentation mode. That is, the RSVP procedure is an atypical way to read text, whereas in our study, participants listened to word sequences much as they would under normal listening conditions (note that the timing of word presentation was very similar in the Mirault et al.^27^ RSVP study—300 ms per word—and in the present study—274 ms per word on average). Therefore, one possible explanation is that RSVP disrupts normal reading behavior, making the reading process harder, and inducing a more careful reading strategy. This would in turn result in effects emerging mainly in errors because RTs would be at ceiling across conditions.

Alternatively, Mirault et al.^27^ tentatively suggested that the different patterns of transposed-word effects seen under parallel and serial word presentation might be due to the relative involvement of bottom-up and top-down processes. Parallel word presentation would be subject to both noisy bottom-up processing, with the noisy association of word identities to specific locations along a line of text^26,34^, as well as top-down sentence-level constraints imposing a syntactically correct word order^22,23^. Sequential word presentation would reduce the noisy bottom-up component since presenting one word after the other would greatly reduce uncertainty in word order, hence leaving top-down constraints as the main source of transposed-word effects. Under sequential presentation conditions participants would therefore be induced into reading the transposed-word sequences as being grammatically correct, hence the effects being seen only in error rates. However, the present findings are problematic for this account since one would expect bottom-up word order information to be relatively unambiguous with sequences of spoken words.

**Figure 2:**
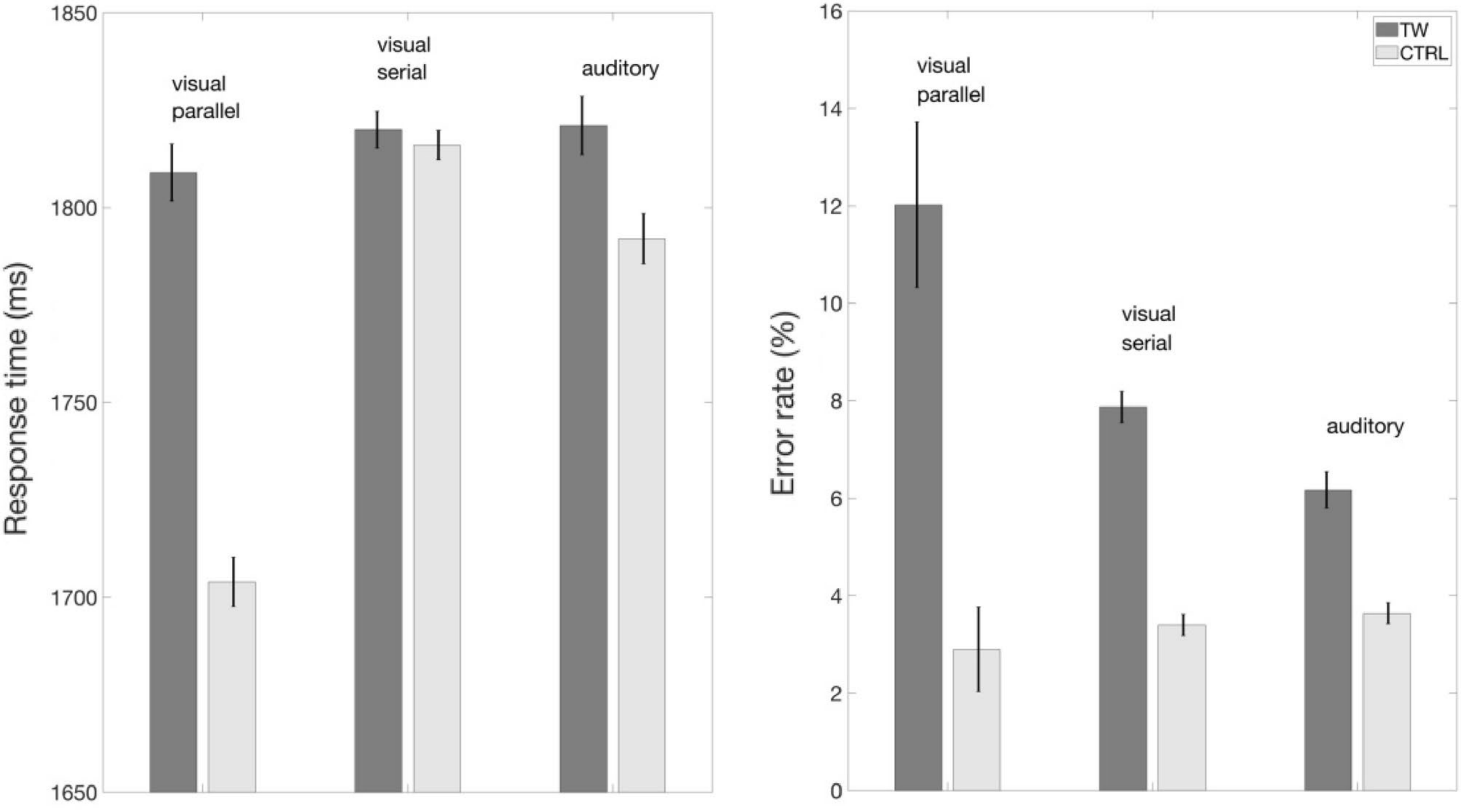
Comparison of transposed-word effects (transposed-word—TW vs. control—CTRL) obtained under parallel presentation of written words (visual parallel: Mirault et al., 2018), serial presentation of written words (visual serial: Mirault et al., 2022), and in the present study (auditory).

The comparison of transposed-word effects across studies provides an important clue as to why the odd-man out is the serial RSVP experiment (Mirault et al.,^27^, Experiment 5). Although not shown in Fig. 2, this is the only experiment to show faster RTs to the ungrammatical sequences than to the grammatically correct sentences. It therefore seems likely that participants in that experiment were detecting the ungrammaticality during the RSVP sequence and responding before the end of the stream. Why were participants in the present study not applying the same strategy? Most likely because of the more naturalistic presentation of stimuli in the present experiment compared with the unnatural presentation of stimuli in RSVP. In other words, participants in the present study were listening to spoken word sequences much like they do during everyday spoken language comprehension, and participants in the Mirault et al.^15^ were reading sequences of written words much like they would normally read text. This explains why we see the same pattern of transposed-word effects in these two studies. The fact that the speech signal provides less ambiguous bottom-up information about word order further explains why the effects are smaller with speech compared with text (see Fig. 2).

In the Introduction we mentioned the “noisy channel” account of how readers / listeners are able to interpret word sequences that include syntactic errors including word transpositions (e.g.,^9^). It is important to note that the vast majority of studies on which this theoretical account is based used written materials, and it will therefore be interesting to know if this approach, and recent developments of the approach (e.g.,^13^) can handle sentence processing in the auditory modality. Although other forms of noise might increase with auditory compared with visual input, we would argue that this is not the case concerning the computation of word order, and on the contrary, word order information should be less noisy with speech than with text. Nevertheless, the general idea that adult readers / listeners are trying to make sense of noisy linguistic input provides a good account of transposed-word effects in general and fits well with the finding that the effects are stronger with more natural presentation conditions, since these would more strongly engage interpretative processes.

Finally, one overarching modality-independent mechanism might well be at play in driving the transposed-word effects seen with both written and spoken language. As mentioned in the Introduction, that is the way in which semantic processing might help compensate for errors in word order. Assuming that the processing of semantic information is less affected than syntactic processing by small changes in word order, this would provide a means to recover a plausible interpretation of a sequence of words and therefore to bias grammatical decisions towards a “yes” response (see Massol et al.,^35^, for a study on the relative contribution of syntax and semantics to the sentence superiority effect). This is clearly an avenue for future research examining effects of word order changes, and other types of ungrammaticalities, on performance in the grammatical decision task with written and spoken sequences of words.

In sum, we tested the same stimuli as Mirault et al. ^15^ in a grammatical decision task (decide as rapidly and as accurately as possible is a sequence of words is grammatically correct or not), but this time with auditory presentation of the word sequences. We found the same pattern of effects as in the original Mirault et al. study, that is that ungrammatical sequences formed by transposing two words in a correct sentence were harder to reject (longer RTs and more errors) compared with ungrammatical sequences that could not be resolved into a correct sentence by transposing any two words. This is the first demonstration of a transposed-word effect in speeded grammatical decisions to speech, and further demonstrates the key role played by syntactic and possibly semantic constraints in generating transposed-word effects in general. Moreover, our results attest to the viability of the grammatical decision task as a tool for examining basic mechanisms in spoken language comprehension, and we expect this task to become as popular as its word-level equivalent—the lexical decision task.

## Data Availability

Datasets and scripts for analyses are available on the OSF website at https://osf.io/9k3sf/.
